# Supplementary material for: ATPase activity of the DEAD-box protein Dhh1 controls processing body formation
Source: eLife. 2016 Oct 3;5:e18746. doi: 10.7554/eLife.18746 (PMC5096884; doi:10.7554/eLife.18746)
Supplement: Supplementary file 1. — DOI: http://dx.doi.org/10.7554/eLife.18746.026 [file elife-18746-supp1.docx]

**SUPPLEMENTARY TABLE S1**

**Yeast strains in this study**

| **Strain #** | **Genotype** |
| --- | --- |
| KWY165 | *MAT a ade2-1 can1-100 GAL phi+ his3-11,15 ura3-1 leu2-3, 112 trp1-1* |
| KWY2379 | *MAT a ade2-1 can1-100 GAL phi+ his3-11,15 ura3-1 leu2-3, 112 trp1-1 dcp2::DCP2-GFP-HisMX6* |
| KWY3238 | *MAT alpha ade2-1 can1-100 GAL phi+ his3-11,15 ura3-1 leu2-3, 112 trp1-1 dhh1∆::KanMX6 dcp2::DCP2-mCherry-NatMX6 pDHH1-GFP-URA3* |
| KWY3239 | *MAT alpha ade2-1 can1-100 GAL phi+ his3-11,15 ura3-1 leu2-3, 112 trp1-1 dhh1∆::KanMX6 dcp2::DCP2-mCherry-NatMX6 pDHH1^DQAD^-GFP-URA3* |
| KWY3264 | *MAT a ade2-1 can1-100 GAL phi+ his3-11,15 ura3-1 leu2-3, 112 trp1-1 dhh1∆::KanMX6 dcp2::DCP2-mCherry-NatMX6 xrn1::XRN1-GFP-HisMX6* |
| KWY3270 | *MAT a ade2-1 can1-100 GAL phi+ his3-11,15 ura3-1 leu2-3, 112 trp1-1 dhh1∆::KanMX6 dcp2::DCP2-mCherry-NatMX6 dcp1::DCP1-GFP-HisMX6* |
| KWY3304 | *MAT a ade2-1 can1-100 GAL phi+ his3-11,15 ura3-1 leu2-3, 112 trp1-1 dhh1∆::KanMX6 dcp2::DCP2-mCherry-NatMX6 edc3::EDC3-GFP-HisMX6* |
| KWY3318 | *MAT a ade2-1 can1-100 GAL phi+ his3-11,15 ura3-1 leu2-3, 112 trp1-1 dhh1∆::KanMX6 dcp2::DCP2-GFP-HisMX6* |
| KWY4901 | *MAT alpha ade2-1 can1-100 GAL phi+ his3-11,15 ura3-1 leu2-3, 112 trp1-1 dhh1∆::KanMX6 dcp2::DCP2-mCherry-NatMX6 pDHH1^5X-Not^-GFP-URA3* |
| KWY5020 | *MAT a ade2-1 can1-100 GAL phi+ his3-11,15 ura3-1 leu2-3, 112 trp1-1 dhh1∆::KanMX6 not1::NOT1-3HA-HisMX6 pDHH1^5X-Not^-TAP-URA3* |
| KWY5058 | *MAT a ade2-1 can1-100 GAL phi+ his3-11,15 ura3-1 leu2-3, 112 trp1-1 dhh1∆::KanMX6 not1::NOT1-3HA-HisMX6 pDHH1-TAP-URA3* |
| KWY5060 | *MAT a ade2-1 can1-100 GAL phi+ his3-11,15 ura3-1 leu2-3, 112 trp1-1 dhh1∆::KanMX6 not1::NOT1-3HA-HisMX6 pDHH1^Q-motif^-TAP-URA3* |
| KWY5129 | *MAT alpha ade2-1 can1-100 GAL phi+ his3-11,15 ura3-1 leu2-3, 112 trp1-1 fba1::FBA1-FLAG-PP7CP-NatMX6 pDHH1^DQAD^-6XHIS-PP7CP-URA3* |
| KWY5131 | *MAT alpha ade2-1 can1-100 GAL phi+ his3-11,15 ura3-1 leu2-3, 112 trp1-1 fba1::FBA1-FLAG-PP7CP-NatMX6 pDHH1^3X-RNA^-6XHIS-PP7CP-URA3* |
| KWY5133 | *MAT alpha ade2-1 can1-100 GAL phi+ his3-11,15 ura3-1 leu2-3, 112 trp1-1 fba1::FBA1-FLAG-PP7CP-NatMX6 pDHH1^DQAD/3X-RNA^-6XHIS-PP7CP-URA3* |
| KWY5135 | *MAT alpha ade2-1 can1-100 GAL phi+ his3-11,15 ura3-1 leu2-3, 112 trp1-1 dhh1∆::NatMX6 fba1::FBA1-FLAG-PP7CP-KanMX6 pDHH1^DQAD^-6XHIS-PP7CP-URA3* |
| KWY5137 | *MAT alpha ade2-1 can1-100 GAL phi+ his3-11,15 ura3-1 leu2-3, 112 trp1-1 dhh1∆::NatMX6 fba1::FBA1-FLAG-PP7CP-KanMX6 pDHH1^3X-RNA^-6XHIS-PP7CP-URA3* |
| KWY5139 | *MAT alpha ade2-1 can1-100 GAL phi+ his3-11,15 ura3-1 leu2-3, 112 trp1-1 dhh1∆::NatMX6 fba1::FBA1-FLAG-PP7CP-KanMX6 pDHH1^DQAD/3X-RNA^-6XHIS-PP7CP-URA3* |
| KWY5170 | *MAT alpha ade2-1 can1-100 GAL phi+ his3-11,15 ura3-1 leu2-3, 112 trp1-1 dhh1∆::KanMX6 dcp2::DCP2-mCherry-NatMX6 pDHH1^DQAD/3X-RNA^-GFP-URA3* |
| KWY5209 | *MAT alpha ade2-1 can1-100 GAL phi+ his3-11,15 ura3-1 leu2-3, 112 trp1-1 dhh1∆::KanMX6 dcp2::DCP2-mCherry-NatMX6 pDHH1^5X-Not/3X-RNA^-GFP-URA3* |
| KWY5242 | *MAT alpha ade2-1 can1-100 GAL phi+ his3-11,15 ura3-1 leu2-3, 112 trp1-1 dhh1∆::KanMX6 dcp2::DCP2-mCherry-NatMX6 pDHH1^3X-RNA^-GFP-URA3* |
| KWY5244 | *MAT alpha ade2-1 can1-100 GAL phi+ his3-11,15 ura3-1 leu2-3, 112 trp1-1 dhh1∆::KanMX6 dcp2::DCP2-mCherry-NatMX6 pDHH1^Q-motif^-GFP-URA3* |
| KWY5246 | *MAT alpha ade2-1 can1-100 GAL phi+ his3-11,15 ura3-1 leu2-3, 112 trp1-1 dhh1∆::KanMX6 dcp2::DCP2-mCherry-NatMX6 pGFP-URA3* |
| KWY5336 | *MAT alpha ade2-1 can1-100 GAL phi+ his3-11,15 ura3-1 leu2-3, 112 trp1-1 dhh1∆::NatMX6 fba1::FBA1-FLAG-PP7L::KanMX6 pGFP-6XHIS-PP7CP-URA3* |
| KWY5338 | *MAT alpha ade2-1 can1-100 GAL phi+ his3-11,15 ura3-1 leu2-3, 112 trp1-1 dhh1∆::NatMX6 fba1::FBA1-FLAG-PP7L::KanMX6 pDHH1-6XHIS-PP7CP-URA3* |
| KWY5340 | *MAT alpha ade2-1 can1-100 GAL phi+ his3-11,15 ura3-1 leu2-3, 112 trp1-1 dhh1∆::NatMX6 fba1::FBA1-FLAG-PP7L::KanMX6 pDHH1^Q-motif^-6XHIS-PP7CP-URA3* |
| KWY5344 | *MAT alpha ade2-1 can1-100 GAL phi+ his3-11,15 ura3-1 leu2-3, 112 trp1-1 dhh1∆::NatMX6 fba1::FBA1-FLAG-PP7L::KanMX6 pGFP-PP7CP-URA3* |
| KWY5346 | *MAT alpha ade2-1 can1-100 GAL phi+ his3-11,15 ura3-1 leu2-3, 112 trp1-1 dhh1∆::NatMX6 fba1::FBA1-FLAG-PP7L::KanMX6 pDHH1-6XHIS-PP7CP-URA3* |
| KWY5618 | *MAT alpha ade2-1 can1-100 GAL phi+ his3-11,15 ura3-1 leu2-3, 112 trp1-1 dhh1∆::NatMX6 fba1::FBA1-FLAG-PP7L::KanMX6 pDHH1^5X-Not^-6XHIS-PP7CP-URA3* |
| KWY5701 | *MAT alpha ade2-1 can1-100 GAL phi+ his3-11,15 ura3-1 leu2-3, 112 trp1-1 dhh1::DHH1^5X-Not^ dcp2::DCP2-mCherry-NatMX6 xrn1::XRN1-GFP-HisMX6* |
| KWY5707 | *MAT a ade2-1 can1-100 GAL phi+ his3-11,15 ura3-1 leu2-3, 112 trp1-1 dhh1::DHH1^5X-Not^ dcp2::DCP2-mCherry-NatMX6 dcp1::DCP1-GFP-HisMX6* |
| KWY5710 | *MAT a ade2-1 can1-100 GAL phi+ his3-11,15 ura3-1 leu2-3, 112 trp1-1 dhh1::DHH1^5X-Not^ dcp2::DCP2-mCherry-NatMX6 edc3::EDC3-GFP-HisMX6* |
| KWY5838 | *MAT a ade2-1 can1-100 GAL phi+ his3-11,15 ura3-1 leu2-3, 112 trp1-1 dcp2::DCP2-mCherry-NatMX6 xrn1::XRN1-GFP-HisMX6* |
| KWY5848 | *MAT a ade2-1 can1-100 GAL phi+ his3-11,15 ura3-1 leu2-3, 112 trp1-1 dcp2::DCP2-mCherry-NatMX6 dcp1::DCP1-GFP-HisMX6* |
| KWY5852 | *MAT a ade2-1 can1-100 GAL phi+ his3-11,15 ura3-1 leu2-3, 112 trp1-1 dcp2::DCP2-mCherry-NatMX6 edc3::EDC3-GFP-HisMX6* |
| KWY6141 | *MAT a ade2-1 can1-100 GAL phi+ his3-11,15 ura3-1 leu2-3, 112 trp1-1 dcp2::DCP2-GFP-HisMX6, dhh1::DHH1^DQAD^* |
| KWY6142 | *MAT alpha ade2-1 can1-100 GAL phi+ his3-11,15 ura3-1 leu2-3, 112 trp1-1 dcp2::DCP2-GFP-HisMX6 xrn1∆::KanMX6* |
| KWY6186 | *MAT alpha ade2-1 can1-100 GAL phi+ his3-11,15 ura3-1 trp1-1 leu2-3, 112∆::CG-LEU2-NOT1-TAP not1∆::TRP1 dcp1::DCP1-GFP-HisMX6* |
| KWY6190 | *MAT alpha ade2-1 can1-100 GAL phi+ his3-11,15 ura3-1 trp1-1 leu2-3, 112∆::CG-LEU2-NOT1^9X-Dhh1^-TAP not1∆::TRP1 dcp1::DCP1-GFP-HisMX6* |
| KWY6540 | *MAT a ade2-1 can1-100 GAL phi+ his3-11,15 ura3-1 trp1-1 leu2-3, 112∆::CG-LEU2-NOT1-TAP , not1∆::TRP1 dhh1::DHH1-GFP-URA3 dcp2::DCP2-mCherry-NatMX6* |
| KWY6541 | *MAT a ade2-1 can1-100 GAL phi+ his3-11,15 ura3-1 trp1-1 leu2-3, 112∆::CG-LEU2-NOT1^9X-Dhh1^-TAP , not1∆::TRP1 dhh1::DHH1-GFP-URA3 dcp2::DCP2-mCherry-NatMX6* |
| KWY6543 | *MAT a ade2-1 can1-100 GAL phi+ his3-11,15 ura3-1 trp1-1 leu2-3, 112∆::CG-LEU2-NOT1-TAP not1∆::TRP1 xrn1::XRN1-GFP-HisMX6 dcp2::DCP2-mCherry-NatMX6* |
| KWY6545 | *MAT a ade2-1 can1-100 GAL phi+ his3-11,15 ura3-1 trp1-1 leu2-3, 112∆::CG-LEU2-NOT1^9X-Dhh1^-TAP not1∆::TRP1 xrn1::XRN1-GFP-HisMX6 dcp2::DCP2-mCherry-NatMX6* |
| KWY6547 | *MAT a ade2-1 can1-100 GAL phi+ his3-11,15 ura3-1 trp1-1 leu2-3, 112∆::CG-LEU2-NOT1-TAP not1∆::TRP1 edc3::EDC3-GFP-HisMX6 dcp2::DCP2-mCherry-NatMX6* |
| KWY6549 | *MAT a ade2-1 can1-100 GAL phi+ his3-11,15 ura3-1 trp1-1 leu2-3, 112∆::CG-LEU2-NOT1^9X-Dhh1^-TAP not1∆::TRP1 edc3::EDC3-GFP-HisMX6 dcp2::DCP2-mCherry-NatMX6* |
| KWY6550 | *MAT alpha ade2-1 can1-100 GAL phi+ his3-11,15 ura3-1 leu2-3, 112 trp1-1 pDHH1-GFP-URA3 dcp2::DCP2-mCherry-NatMX6* |
| KWY6551 | *MAT alpha ade2-1 can1-100 GAL phi+ his3-11,15 ura3-1 leu2-3, 112 trp1-1 pDHH1^DQAD^-GFP-URA3 dcp2::DCP2-mCherry-NatMX6* |
| KWY6554 | *MAT a ade2-1 can1-100 GAL phi+ his3-11,15 ura3-1 leu2-3, 112 trp1-1 pab1::PAB1-GFP::HisMX6 dcp2::DCP2-mCherry-NatMX6* |
| KWY6555 | *MAT a ade2-1 can1-100 GAL phi+ his3-11,15 ura3-1 leu2-3, 112 trp1-1 dhh1::DHH1^DQAD^ pab1::PAB1-GFP::HisMX6 dcp2::DCP2-mCherry-NatMX6* |
| KWY6837 | *MAT a ade2-1 can1-100 GAL phi+ his3-11,15 ura3-1 leu2-3, 112 trp1-1* dhh1∆::KanMX6 *dcp2::DCP2-mCherry-NatMX6 xrn1::XRN1-GFP-HisMX6 pDHH1-TAP-URA3* |
| KWY6838 | *MAT a ade2-1 can1-100 GAL phi+ his3-11,15 ura3-1 leu2-3, 112 trp1-1* dhh1∆::KanMX6 *dcp2::DCP2-mCherry-NatMX6 xrn1::XRN1-GFP-HisMX6 pDHH1^Q-motif^-TAP-URA3* |
| KWY6839 | *MAT a ade2-1 can1-100 GAL phi+ his3-11,15 ura3-1 leu2-3, 112 trp1-1* dhh1∆::KanMX6 *dcp2::DCP2-mCherry-NatMX6 xrn1::XRN1-GFP-HisMX6 pDHH1^3X-RNA^-TAP-URA3* |
| KWY6840 | *MAT a ade2-1 can1-100 GAL phi+ his3-11,15 ura3-1 leu2-3, 112 trp1-1* dhh1∆::KanMX6 *dcp2::DCP2-mCherry-NatMX6 dcp1::DCP1-GFP-HisMX6 pDHH1-TAP-URA3* |
| KWY6841 | *MAT a ade2-1 can1-100 GAL phi+ his3-11,15 ura3-1 leu2-3, 112 trp1-1* dhh1∆::KanMX6 *dcp2::DCP2-mCherry-NatMX6 dcp1::DCP1-GFP-HisMX6 pDHH1^Q-motif^-TAP-URA3* |
| KWY6842 | *MAT a ade2-1 can1-100 GAL phi+ his3-11,15 ura3-1 leu2-3, 112 trp1-1* dhh1∆::KanMX6 *dcp2::DCP2-mCherry-NatMX6 dcp1::DCP1-GFP-HisMX6 pDHH1^3X-RNA^-TAP-URA3* |
| KWY6843 | *MAT a ade2-1 can1-100 GAL phi+ his3-11,15 ura3-1 leu2-3, 112 trp1-1* dhh1∆::KanMX6 *dcp2::DCP2-mCherry-NatMX6 edc3::EDC3-GFP-HisMX6 pDHH1-TAP-URA3* |
| KWY6844 | *MAT a ade2-1 can1-100 GAL phi+ his3-11,15 ura3-1 leu2-3, 112 trp1-1* dhh1∆::KanMX6 *dcp2::DCP2-mCherry-NatMX6 edc3::EDC3-GFP-HisMX6 pDHH1^Q-motif^-TAP-URA3* |
| KWY6845 | *MAT a ade2-1 can1-100 GAL phi+ his3-11,15 ura3-1 leu2-3, 112 trp1-1* dhh1∆::KanMX6 *dcp2::DCP2-mCherry-NatMX6 edc3::EDC3-GFP-HisMX6 pDHH1^3X-RNA^-TAP-URA3* |
| KWY6846 | *MAT a ade2-1 can1-100 GAL phi+ his3-11,15 ura3-1 leu2-3, 112 trp1-1 DHH1 not1::NOT1-3HA-HisMX6* |
| KWY6847 | *MAT a ade2-1 can1-100 GAL phi+ his3-11,15 ura3-1 leu2-3, 112 trp1-1 dhh1∆::KanMX6 dcp2::DCP2-mCherry-NatMX6 pDHH1^F66R^-GFP-URA3* |
| KWY6848 | *MAT a ade2-1 can1-100 GAL phi+ his3-11,15 ura3-1 leu2-3, 112 trp1-1 dhh1∆::KanMX6 dcp2::DCP2-mCherry-NatMX6 pDHH1^Q73A^-GFP-URA3* |
