# Supplementary material for: ATPase activity of the DEAD-box protein Dhh1 controls processing body formation
Source: eLife. 2016 Oct 3;5:e18746. doi: 10.7554/eLife.18746 (PMC5096884; doi:10.7554/eLife.18746)
Supplement: Supplementary file 2. — (A) List of plasmids used in this study. (B) List of oligos used in this study. Bold text denotes region of homology between genome and oligonucleotide. Italicized text denotes homology between plasmid and oligonucleotide. (C) List of Dhh1 and Not1 mutants used in this study. DOI: http://dx.doi.org/10.7554/eLife.18746.027 [file elife-18746-supp2.docx]

**SUPPLEMENTARY TABLE S2**

**A) Plasmids used in this study**

| **Plasmid #** | **Description** |
| --- | --- |
| pKW1716 | pSV272-GLE1(244-538) |
| pKW2312 | pRS316 *P(DHH1)*-*DHH1-GFP* |
| pKW2313 | pRS316 *P(DHH1)*-*DHH1^DQAD^-GFP* |
| pKW2321 | pRS316 *P(DHH1)*-*DHH1-6xHis-PP7CP* |
| pKW2322 | pRS316 *P(DHH1)*-*DHH1^DQAD^-6xHis-PP7CP* |
| pKW2420 | pRS316 *P(DHH1)-GFP* |
| pKW2421 | pRS316 *P(DHH1)*-*DHH1-CBP-TEV-ZZ* |
| pKW2866 | pRS316 *P(DHH1)*-*DHH1^3X-RNA^-6XHis-PP7CP* |
| pKW2867 | pRS316 *P(DHH1)*-*DHH1^3X-RNA^-GFP* |
| pKW3040 | pRS316 *P(DHH1)*-*DHH1^DQAD/3X-RNA^-GFP* |
| pKW3066 | pRS316 *P(DHH1)-DHH1^F66R^-GFP* |
| pKW3069 | pRS316 *P(DHH1)-DHH1^Q73A^-GFP* |
| pKW3073 | pRS316 *P(DHH1)*-*DHH1^Q-motif^-6xHis-PP7CP* |
| pKW3074 | pRS316 *P(DHH1)*-*DHH1^Q-motif^-CBP-TEV-ZZ* |
| pKW3333 | pRS316 *P(DHH1)*-*DHH1^5X-Not^-GFP* |
| pKW3349 | pRS316 *P(DHH1)*-*DHH1^5X-Not^-CBP-TEV-ZZ* |
| pKW3353 | pRS316 *P(DHH1)*-*DHH1^5X-Not^-6xHis-PP7CP* |
| pKW3469 | pETMCN_*6xHIS_V5_NOT1^MIF4G^* |
| pKW3477 | pRS316 *P(DHH1)*-*DHH1^DQAD/3X-RNA^-6xHis-PP7CP* |
| pKW3631 | pETMCN_*6xHIS_V5_DHH1-mCherry* |
| pKW3632 | pETMCN_*6xHIS_V5_DHH1^DQAD^-mCherry* |
| pKW3714 | pETMCN_*6xHIS_V5_DBP5-mCherry* |
| pKW3772 | pNH605 *P(NOT1)-NOT1^9X-Dhh1^-CBP-TEV-ZZ* |
| pKW3781 | pNH605 *P(NOT1)-NOT1-CBP-TEV-ZZ* |
| pKW4057 | pETMCN_HisV5_Dhh1-F66R_mCherry |
| pKW3639 | pETMCN_HisV5_Dhh1-DQAD_mCherry |

**B) Oligos used in this study**

| **Oligo #** | **Sequence** | **Description** |
| --- | --- | --- |
| UC4904 | **tgatcttttcgtcactgacgggtccctgctattagattt***gggtgctggtttaattaacat* | Edc3, fw; C-terminal GFP tagging |
| UC4905 | **tatacgtatgtatccagtttaggctaaagtaattcttggt***gaattcgagctcgtttaaac* | Edc3, rev; C-terminal GFP tagging |
| UC4907 | **aaaatatcttctggaaaatgagccaaaagattcttttgct***ggtgctggtttaattaacat* | Dcp1, fw; C-terminal GFP tagging |
| UC4908 | **atttaaaaaaaattctcacttgggcatctcacctctgtgc***gaattcgagctcgtttaaac* | Dcp1, rev; C-terminal GFP tagging |
| UC4878 | **caatgctgctgaccgtgataataaaaaagacgaatctact***ggtgctggtttaattaacat* | Xrn1, fw; C-terminal GFP tagging |
| UC4879 | **taaagtaacctcgaatatacttcgtttttagtcgtatgtt***gaattcgagctcgtttaaac* | Xrn1, rev; C-terminal GFP tagging |
| US5019 | tcatgcaagaatgaagcaacaagaagcaaataaagttttccacgaatttcgt | DHH1, fw; mutagenic primer R322A for Dhh1^3X-RNA^ mutant |
| US5020 | acgaaattcgtggaaaactttatttgcttcttgttgcttcattcttgcatga | DHH1, rev; mutagenic primer R322A for Dhh1^3X-RNA^ mutant |
| US5021 | ttcgtacattggtctgcgccgatttattgacccgt | DHH1, fw; mutagenic primer S340A for Dhh1^3X-RNA^ mutant |
| US5022 | acgggtcaataaatcggcgcagaccaatgtacgaa | DHH1, rev; mutagenic primer S340A for Dhh1^3X-RNA^ mutant |
| US5023 | ccaaaacggcagaaacatatttacatgctattggtagatccggtagg | DHH1, fw; mutagenic primer R370A for Dhh1^3X-RNA^ mutant |
| US5024 | cctaccggatctaccaatagcatgtaaatatgtttctgccgttttgg | DHH1, rev; mutagenic primer R370A for Dhh1^3X-RNA^ mutant |
| UC5770 | **ctgaaatcatgattttcgtatataaataaatgcagttttt***gaattcgagctcgtttaaac* | NOT1, fw; C-terminal 3HA tagging |
| UC5852 | gttaatgggtatttttgaagccggtagggaaaaaccatcccctattcaagaag | DHH1, fw; mutagenic primer F66R for Dhh1^Q-motif^ mutant |
| UC5853 | cttcttgaataggggatggtttttccctaccggcttcaaaaatacccattaac | DHH1, rev; mutagenic primer F66R for Dhh1^Q-motif^ mutant |
| UC5854 | gttttgaaaaaccatcccctattgcagaagaggctattccggtag | DHH1, fw; mutagenic primer Q73A for Dhh1^Q-motif^ mutant |
| UC5855 | ctaccggaatagcctcttctgcaataggggatggtttttcaaaac | DHH1, rev; mutagenic primer Q73A for Dhh1^Q-motif^ mutant |
| UC6036 | **caccatcaatagaaggcaaacccctctacaatccaacgca***cggatccccgggttaattaa* | NOT1, rev; C-terminal 3-HA tagging |
| UC6097 | **atcccaggcctaaaatacgacaagaaagaaaatagtagta***atgggttccatcaataataa* | DHH1; fw; For integrating *DHH1* mutants into *dhh1∆* strains |
| UC6098 | **tatctcaccacagtagttattttttcttagatattct***ttaatactggggttgtgactgac* | DHH1; rv; For integrating *DHH1* mutants into *dhh1∆* strains |
| CH393 | tgttcattattcatcatggaccaagccgataaaatgttatctcgt | DHH1, fw; mutagenic primer E195Q for Dhh1^DQAD^ mutant |
| CH394 | acgagataacattttatcggcttggtccatgatgaataatgaaca | DHH1, rev; mutagenic primer E195Q for Dhh1^DQAD^ mutant |
| CH574 | atggtttttcaaaaccggcttcttcaatacccattaacagctctcttttcaaataaaaatc | DHH1, rev; mutagenic primer F62E for Dhh1^5X-Not^ mutant |
| CH575 | cttaaatacaaagggtaatacttttgaagatttttatttgaaagaagagctgttaatgggtattttt | DHH1, fw; mutagenic primer R55E for Dhh1^5X-Not^ mutant |
| CH576 | gatttttatttgaaaagagagctgttaatgggtattgaagaagccggttttgaaaaaccat | DHH1, fw; mutagenic primer F62E for Dhh1^5X-Not^ mutant |
| CH577 | aaaaatacccattaacagctcttctttcaaataaaaatcttcaaaagtattaccctttgtatttaag | DHH1, rev; mutagenic primer R55E for Dhh1^5X-Not^ mutant |
| CH578 | cacggttagtagaattacaaaaaataatggcttgctcaatttcaagcttagagaataaagtatttaaacaatgtagc | DHH1, rev; mutagenic primer Q282E and N284E for Dhh1^5X-Not^ mutant |
| CH579 | gctacattgtttaaatactttattctctaagcttgaaattgagcaagccattattttttgtaattctactaaccgtg | DHH1, fw; mutagenic primer Q282E and N284E for Dhh1^5X-Not^ mutant |
| CH580 | atcggagcagaccaatgtttcaaccttaccttgacgaaattcgtgg | DHH1, rev; mutagenic primer R335E for Dhh1^5X-Not^ mutant |
| CH581 | ccacgaatttcgtcaaggtaaggttgaaacattggtctgctccgat | DHH1, fw; mutagenic primer R335E for Dhh1^5X-Not^ mutant |
| CH1037 | **ttcagggtctaatgaattattaagcattttgcataggaag** *cggatccccgggttaattaa* | Dcp2, fw; C-terminal GFP tagging |
| CH1038 | **catttacagtgtgtctataaaacgtataacacttattctt***gaattcgagctcgtttaaac* | Dcp2, rev; C-terminal GFP tagging |
| CH2445 | **cactattggtaaaagataagcaattgagaaacgtcactcagggacaattgattac***cggatccccgggttaattaa* | NOT1, fw; deletion primer |
| CH2446 | **ttatatttttttttctgaaatcatgattttcgtatataaataaatgcagttttt***gaattcgagctcgtttaaac* | NOT1, rev; deletion primer |

**C) Dhh1 and Not1 mutants used in this study**

| **Mutant Protein** | **Mutation(s)** |
| --- | --- |
| Dhh1^DQAD^ | E195Q |
| Dhh1^Q-motif^ | F66R, Q73A |
| Dhh1^3X-RNA^ | R322A, S340A, R370A |
| Dhh1^5X-Not^ | R55E, F62E, Q282E, N284E, R335E |
| Dhh1^DQAD/3X-RNA^ | E195Q, R322A, S340A, R370A |
| Dhh1^5X-Not/3X-RNA^ | R55E, F62E, Q282E, N284E, R322A, R335E, S340A, R370A |
| Not1^9X-Dhh1^ | F791A, N795A, K804A, E823R, N834A, Y835A, K962A, F967A, E970A |
