# Supplementary material for: ATPase activity of the DEAD-box protein Dhh1 controls processing body formation
Source: eLife. 2016 Oct 3;5:e18746. doi: 10.7554/eLife.18746 (PMC5096884; doi:10.7554/eLife.18746)
Supplement: Supplementray file 3. — DOI: http://dx.doi.org/10.7554/eLife.18746.028 [file elife-18746-fig3.docx]

**SUPPLEMENTARY TABLE S3**

**smFISH probes**

| *GFA1* | 1 tcctctggatctttccac  2 ccatccactaaggtgtcg  3 accggtggaatcatagcc  4 atcagcttcgtcaccatc  5 tagcccatctagtatgcg  6 acttgttctggtcgaccg  7 ggtcttctgggtcagatc  8 ctcggtatcggtatcact  9 agtcatgcccattttgta  10 acccgtatgaaccttcta  11 tcagtaaaggggaccctt  12 ccacatccacgaagtcga  13 ggaatttccggttgacca  14 ggcccaagccaaatgatt  15 gcattttgggaaccagct  16 tcattggcggcaattggt  17 gccctggattgagaatgt  18 aattccaccggtgttgga  19 gatgccgcatccgaagaa  20 gcaccttcttggtatgtt  21 ttgatgcgcctacttctc  22 aaatggtcgtaagggccc  23 agtcgattctacctctca  24 atgcctttaaaccaccca  25 tccgtgctcttctgacaa  26 gtaccgcatgcgatcatg  27 agatagcacgagtagcca  28 agggcattttctgtccag  29 cacgcatacatcgtctct  30 gcagtttcaccactttgt  31 atttagagccagcatggt  32 gttaaggctcctctttct  33 acaccacagtgggtgaca  34 ccaatttcaggaccagcg  35 cacggtcatctgacagcg  36 cttaatttggcccgggat  37 attcttggttccagcttt  38 ttcagtcgcacagagctt  39 attggtaacctctaccca  40 aaagcaccttccagagca  41 cctgccaaaacaccttca  42 gacaccgtgcttcaactc  43 agttttcgtccaccaagg  44 agagagtctctggtacca  45 aattggatggccctttct  46 tagatttttgcgcccaca  47 ggtttgcaggtcgattga  48 tccctttattaacagcca |
| --- | --- |
| *FBA1* | 1 agatttgttcaacaccca  2 acaccggtctttctcttt  3 gttgtggacatcttcacc  4 acttgtgttccttagcgt  5 ggtgacgttaatagctgg  6 aagcagcgacggcagtag  7 attggggacttgctgtct  8 caccgttagaggtttgca  9 ccagcgaagtaagcagca  10 ccttcgttagagataccc  11 cacccttgatggaagcat  12 tagtgggcagcggcaata  13 gctggagcaatggatctg  14 agacaactgggataccgt  15 acttcttggcacagtggt  16 taccatcgaaccatggca  17 gcttcatcagcttccaac  18 aatggttcaccgtgttcc  19 ccaacatgtgggaggaga  20 tcttcatcggtttcttca  21 agcggccattctcttgaa  22 tttctaaccattggtcca  23 ccggtaataccgatttcc  24 aacaccatcttcttcacc  25 cttccttgtcagcgtttt  26 gttctggcttggtgtaca  27 agccttgtagacgttgta  28 ttggagagattgggtgca  29 gttaccgaaagcagcagc  30 ccagcgtacaaaccgtga  31 ctggtctcaaagcgatgt  32 acttttggtgttcagcca  33 accaacttgttctctggt  34 atggcttttcttccttgc  35 ccaccgtggaagaccaag  36 cttggacagtagaaccgg  37 accgttgtcaataccagt  38 tccaagttgaccttgaca  39 agcgtattgacagtcagt  40 agtctctgataccagtca  41 gtccttcttgttcaagac  42 ccgactggggacattatg  43 ttctggaccttctgggtt  44 agaacttcttgtttggct  45 aacccagactcttgggtc  46 tggtcttttcaccttctc  47 ttggtgatcttagcaccc  48 gtggtacggaaagtttcc |
| *PAT1* | 1 ctcttcaaagtccagaggac  2 aatagtcgttctcctcaagt  3 tcaccaaatgtttcgtcgtt  4 aagtcggtaccaacctgaac  5 ctgctgtgaggatttccaaa  6 gcagtagctgcaacatatga  7 tggtgcagtagaccacaaag  8 tcgacaagattggctgtagg  9 tgttgtctttccaagtcttg  10 ccatggcgtggaaattcata  11 gcattggaaattgctgctga  12 aaattgtgagggacctggtt  13 aacttgtacaggacccattg  14 ggtgaaggcgaagcttgaac  15 aaggagtgttggacataccg  16 ctttgcagaggcattttagt  17 caggcgacaaatctctcttc  18 atctgcaaacgtctttgctc  19 aagattttctccactttggc  20 aagtccttatcacgaggagt  21 taagggtcctcagtgacaat  22 cgcctctttggataatcttg  23 ttgttggattcggacgtgat  24 atacgccctagcaatcaaac  25 ggcaatatcggttctcttat  26 agccttttctacttgacttt  27 tgtttgcttgcttattatcc  28 ggaagagattttgcccagaa  29 cttggattcttgctgttcaa  30 ttggtcagagagtcagtgac  31 tgcttagaagcaacagcagc  32 caccattaccgttgttgaac  33 ccccagatttgttcaaattt  34 tcaagttagcttccaagtct  35 caatttttgctgcttatcca  36 ggtgtagtcttgtaggaact  37 ctgtagcaacagacccataa  38 ccaattttggaggtggtcaa  39 ttgcttgattaatgcggcac  40 ctcattccatgtggagattt  41 cagctgaatcttactttcca  42 aacgcatgatgtggtcgtta  43 actgccaaatgtatgcttca  44 gctttccacttagtgctaaa  45 tttcttttgtaaggtctccg  46 cctctgaggtaatacactca  47 accccataacgtttaggaac  48 ttctgatatttcaccatcgc |
| *PGK1* | 1 gtccaaatcttggacagaca  2 atgaagacacgcttgtcctt  3 tgggacgttgaagtcaactc  4 gaagtgatcttcttaccgtc  5 agcagcaacaattctttggt  6 aaacgtacttgatggttggc  7 agacaacgtatcttgggtgg  8 tggtctacccaagtgagaag  9 atttttcgtttctttcaccg  10 aattccttagcaactggagc  11 gacatccttacccaacaatg  12 ccgacacagtcgttcaagaa  13 ttgacagcggcttcaacttc  14 aaataacggaacctggggca  15 tggtaacgcaagttttccaa  16 ttctggaaccttcttcttcg  17 ttgaccttttgaccatcgac  18 cttttgaacatcttccttgg  19 cagccaaagagctcaattcg  20 taccgaaggcatcgttgatg  21 gaagagtgagctctgtgagc  22 tgtggcaagtcgaaaccgac  23 caacaagaaaccggcagcac  24 accgaagtacttcaattcct  25 tctggttgggttctccaaag  26 cacctaagatggccaagaat  27 aatcttgtcagcaaccttgg  28 cgaccttgtccaacaagttg  29 ccaccaccaatgatgataga  30 cttcttgaaggtgaaagcca  31 ccgatttcagtgttttccaa  32 cagccttgtcgaagatggag  33 ccttttccatcaactttgga  34 gaagtcgactggcaagacga  35 cagcagagaaagcatcagca  36 agtgacagtcttggtgttgg  37 cagctggaataccttccttg  38 ctagattctggaccattgtc  39 tgcaacagtagcagcaaaca  40 ttccagacaatggtcttagc  41 ttcgaaaacacctggtggac  42 aacaaagccttagtaccagc  43 agagctcttgacaacttcgt  44 caccaatgatgacggtgtta  45 cgtacttcttagcgacagtg  46 atgggagatcttgtcagtga  47 aattccaaagaagcaccacc  48 aacacctggcaattccttac |
